# ARID1A protein expression is retained in ovarian endometriosis with *ARID1A* loss-of-function mutations: implication for the two-hit hypothesis

**DOI:** 10.1038/s41598-020-71273-7

**Published:** 2020-08-31

**Authors:** Nozomi Yachida, Kosuke Yoshihara, Kazuaki Suda, Hirofumi Nakaoka, Haruka Ueda, Kentaro Sugino, Manako Yamaguchi, Yutaro Mori, Kaoru Yamawaki, Ryo Tamura, Tatsuya Ishiguro, Masanori Isobe, Teiichi Motoyama, Ituro Inoue, Takayuki Enomoto

**Affiliations:** 1 Department of Obstetrics and Gynecology, Niigata University Graduate School of Medical and Dental Sciences, 1-757 Asahimachi-dori, Chuo-ward, Niigata, 951-8510 Japan; 2 Human Genetics Laboratory, National Institute of Genetics, Mishima, 411-8540 Japan; 3 Department of Cancer Genome Research, Sasaki Institute, Sasaki Foundation, Chiyoda-ku, Tokyo, 101-0062 Japan; 4 Department of Molecular and Diagnostic Pathology, Niigata University Graduate School of Medical and Dental Sciences, Niigata, 951-8510 Japan

**Keywords:** Ovarian cancer, Cancer genomics, Oncogenesis

## Abstract

*ARID1A* loss-of-function mutation accompanied by a loss of ARID1A protein expression is considered one of the most important driver events in endometriosis-associated ovarian cancer. Although our recent genomic study clarified that *ARID1A* loss-of-function mutations were detected in 13% of ovarian endometriosis, an association between the *ARID1A* mutation status and ARID1A protein expression in ovarian endometriosis remains unclear. We performed immunohistochemical staining for ARID1A in 78 ovarian endometriosis samples and 99 clear cell carcinoma samples. We revealed that not only 70 endometriosis samples without *ARID1A* mutations but also eight endometriosis samples with *ARID1A* loss-of-function mutations retained ARID1A protein expression. On the other hand, most of clear cell carcinomas with *ARID1A* loss-of-function mutations showed a loss of ARID1A protein expression. In particular, clear cell carcinoma samples which harbor multiple *ARID1A* loss-of-function mutations or both a single *ARID1A* loss-of-function mutation and *ARID1A* allelic imbalance lost ARID1A protein expression. However, ARID1A protein expression was retained in seven clear cell carcinomas with *ARID1A* loss-of-function mutations. These results suggest that a single *ARID1A* loss-of-function mutation is insufficient for ARID1A loss in ovarian endometriosis and some clear cell carcinoma. Further driver events may be needed for the malignant transformation of ovarian endometriosis with *ARID1A* loss-of-function mutations.

## Introduction

The AT-rich interaction domain 1A (*ARID1A*) gene is located on chromosome 1p36.11 and encodes ARID1A*,* a key component of the SWI/SNF complex^1^. The SWI/SNF complex plays an important role in chromatin remodeling and is associated with numerous biological functions, such as differentiation and proliferation^2^. Therefore, aberrations in the SWI/SNF complex subunits have the potential to cause cancer. In particular, *ARID1A*, which is well known as a tumor suppressor gene, is frequently mutated in a wide variety of cancers^3,4^. COSMIC data demonstrated that more than half of *ARID1A* mutations are loss-of-function mutations, including frameshift indels mutations, and nonsense mutations, that lead to a loss of ARID1A protein expression in cancer cells^5^.

*ARID1A* mutation is considered one of the most important driver events in endometriosis-associated ovarian cancer^6–8^. According to previous studies, including ours^6,7,9–13^, 46–70% of clear cell carcinomas and 30–46% of endometrioid carcinomas harbor *ARID1A* mutations, and immunohistochemical analysis has demonstrated that *ARID1A* loss-of-function mutations are strongly correlated with the loss of ARID1A protein expression in endometriosis-associated ovarian cancer^6,7^. On the other hand, our recent genomic study clarified that *ARID1A* loss-of-function mutations are detected in 13% of ovarian endometriosis cases^14^. Some deep infiltrating endometriosis cases also harbor *ARID1A* mutations^15,16^. Although several previous studies demonstrated that ARID1A was expressed in endometriosis by immunohistochemical analysis^17–22^, the mutation status of *ARID1A* in endometriotic epithelial cells was not investigated. The association of ARID1A protein expression with *ARID1A* mutations in benign endometriosis remains unclear.

In this study, we performed immunohistochemical staining for ARID1A in ovarian endometriosis samples whose *ARID1A* mutation status was determined by whole-exome sequencing or target gene sequencing to clarify the correlation between ARID1A protein expression and the *ARID1A* mutation status in ovarian endometriosis. Additionally, we evaluated an association between ARID1A protein expression and the *ARID1A* mutation status in ovarian clear cell carcinomas by immunohistochemical analysis. We demonstrated that ARID1A protein expression was retained in all ovarian endometriosis samples and a small portion of ovarian clear cell carcinoma samples harboring *ARID1A* loss-of-function mutations.

## Results

### ARID1A protein expression in ovarian endometriosis

**Figure 1:**
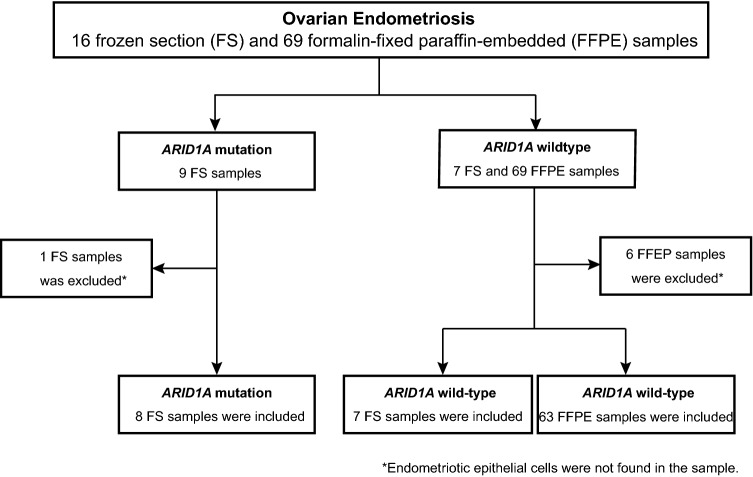
Final analysis set of ovarian endometriosis in this study. We recruited 54 ovarian endometriosis patients for which whole-exome sequencing or target gene sequencing was conducted in our previous study^14^. Then, we collected 16 frozen tissue section samples from 7 patients with *ARID1A* mutations and 69 formalin-fixed paraffin-embedded (FFPE) samples from 47 patients without *ARID1A* mutations.

**Figure 2:**
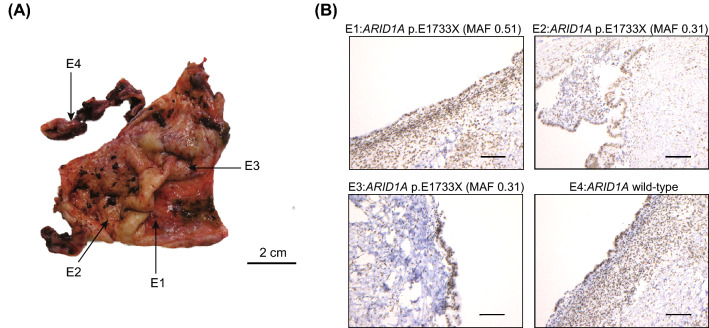
ARID1A protein expression in ovarian endometriosis (ENDO_3). **(A)** Multisampling sites in a unilateral ovarian endometriosis lesion are shown. **(B)** ARID1A protein expression was observed in not only one *ARID1A* wild-type sample but also three *ARID1A* p.E1733X samples. The scale bars represent 100 µm.

We assessed ARID1A immunoreactivity in 15 frozen section samples derived from six ovarian endometriosis patients (Fig. 1). We performed multiregional sampling from ovarian endometriosis tissues in three patients (Table 1). A representative image of ARID1A immunostaining for a multisampling case (ENDO_3) is shown in Fig. 2. Both the *ARID1A* wild-type region and the *ARID1A* mutated regions showed positive immunoreactivity for ARID1A. Immunohistochemical analysis demonstrated positive immunoreactivity for ARID1A in all eight frozen section samples harboring *ARID1A* loss-of-function mutations and seven frozen section samples without *ARID1A* mutations. Table 1 shows the mutation status of other cancer-associated genes in samples with *ARID1A* mutations. While mutations in oncogenes such as *PIK3CA* and *KRAS* were detected in two samples (ENDO1 and ENDO3), mutations in tumor suppressor genes such as *PTEN*, *ATM*, and *TP53* were not detected in any of the 15 ovarian endometriosis samples.

**Table 1:** List of ovarian endometriosis samples showing the *ARID1A* mutation status and ARID1A protein expression.

Patient	Sampling site	*ARID1A* mutation	MAF	*PIK3CA* mutation	MAF	*KRAS* mutation	MAF	ARID1A protein expression
ENDO_1	E1	p.G1711fs	0.46	p.C378F	0.37	p.Q61H	0.45	Positive
ENDO_2	E1	WT						Positive
	E2	Q537X	0.25					Positive
	E3	WT						Positive
	E4	WT						Positive
ENDO_3	E1	E1733X	0.51	p.G118D	0.36	p.G12D	0.44	Positive
	E2	E1733X	0.31	p.G118D	0.48	p.G12D	0.33	Positive
	E3	E1733X	0.31	p.G118D	0.39	p.G12D	0.30	Positive
	E4	WT						Positive
ENDO_4	E1	S825fs	0.37					Positive
ENDO_5	E1	Q1493X	0.34					Positive
	E2	WT						Positive
	E3	WT						Positive
	E4	WT						Positive
ENDO_6	E1	p.F2208fs	0.42					Positive

Next, we assessed ARID1A protein expression in 63 FFPE samples derived from 41 ovarian endometriosis patients without *ARID1A* mutations (Fig. 1). As expected, ARID1A protein expression was detected in all 63 FFPE tissue samples. In summary, all 78 endometriosis samples retained ARID1A protein expression regardless of the *ARID1A* mutation status.

To clarify the significance of *ARID1A* loss-of-function mutations in endometriosis, we compared the clinicopathological features of ovarian endometriosis patients with *ARID1A* loss-of-function mutations to those without *ARID1A* mutations (Supplementary Table 1). Interestingly, ovarian endometriosis patients with *ARID1A* loss-of-function mutations had a higher frequency of endometriosis lesions in bilateral ovaries (*P* = 0.006). There were no differences in other characteristics according to the *ARID1A* mutation status (Supplementary Table 1).

### Correlation between ARID1A protein expression and the *ARID1A* mutation status in ovarian clear cell carcinoma

**Figure 3:**
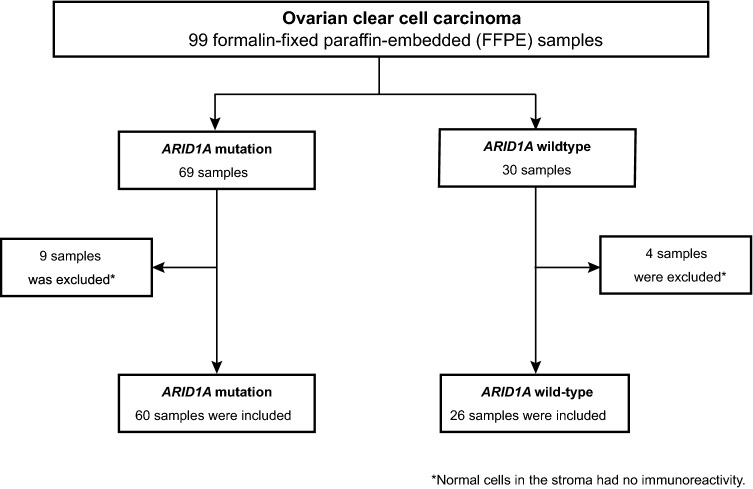
Final analysis set of ovarian clear cell carcinoma in this study. We enrolled 99 patients with ovarian clear cell carcinoma which were already sequenced in our previous study^12^. Additionally, we prepared FFPE tissue sections from 99 clear cell carcinoma cases for immunohistochemical analysis.

**Figure 4:**
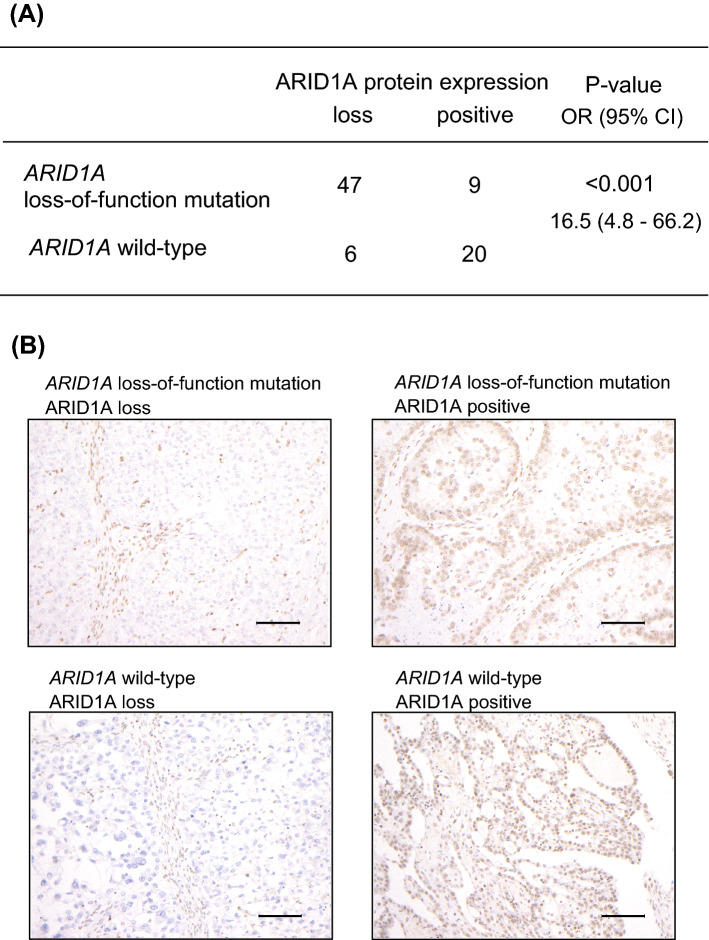
The association between *ARID1A* loss-of-function mutations and ARID1A protein expression in ovarian clear cell carcinomas. **(A)** The number of clear cell carcinomas with or without ARID1A protein expression and/or *ARID1A* mutations is shown. **(B)** Representative ARID1A staining images correspond to four patterns on the basis of *ARID1A* mutations and ARID1A protein expression. The scale bars represent 100 µm.

To evaluate an association between ARID1A protein expression and the *ARID1A* mutation status, we performed immunohistochemical analysis for 99 ovarian clear cell carcinoma samples whose *ARID1A* mutation status was already investigated in our previous study (Supplementary Table 2) ^12^. Nine clear cell carcinomas with *ARID1A* mutations and four clear cell carcinomas without *ARID1A* mutations were excluded from this analysis because of the low antigenicity or low quality of FFPE section samples (Fig. 3). Of 60 clear cell carcinoma samples with *ARID1A* mutations, 49 (81.7%) showed a loss of ARID1A protein expression (Table 2). Specifically, 47 of 56 samples (83.9%) with *ARID1A* loss-of-function mutations showed a loss of ARID1A protein expression. On the other hand, 6 of 26 samples (23.1%) without *ARID1A* mutations also demonstrated a loss of ARID1A protein expression. The presence of *ARID1A* loss-of-function mutations was significantly associated with the loss of ARID1A protein expression in clear cell carcinomas (*P* < 0.001) (Fig. 4A) and the representative ARID1A staining images correspond to four patterns on the basis of *ARID1A* mutations and ARID1A protein expression (Fig. 4B).

**Table 2:** Association of ARID1A protein expression with the *ARID1A* mutation pattern in ovarian clear cell carcinoma.

*ARID1A* mutation pattern	Number	ARID1A protein expression	
		Loss	Positive
Two or more nonsense and/or indel mutations	15	14	1
One nonsense or one indel mutation	40	32	8
One indel mutation and one silent mutation	1	1	0
One missense mutation	1	0	1
One splicing mutation	3	2	1
Total	60	49	11

**Figure 5:**
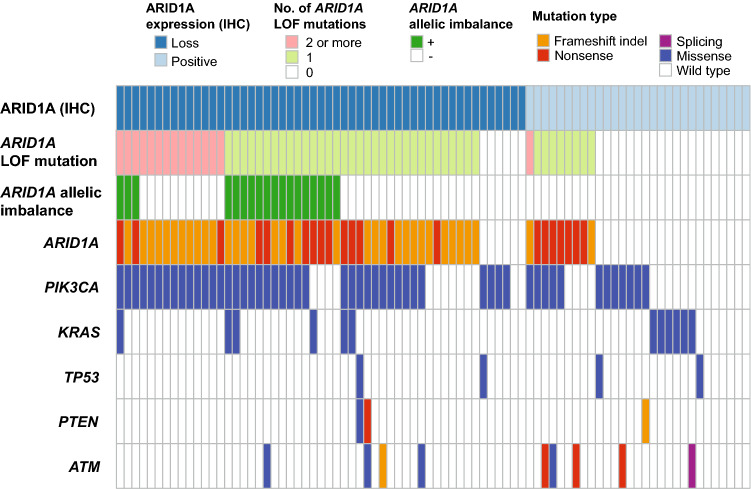
Strong correlation between *ARID1A* allelic imbalance or the number of *ARID1A* loss-of-function mutations and ARID1A protein expression in clear cell carcinoma. The heatmap shows the landscape of the *ARID1A* mutation status, allelic imbalance, ARID1A protein expression and other cancer-associated gene mutations in each clear cell carcinoma sample.

We examined the correlation of *ARID1A* loss-of-function mutations with the loss of ARID1A protein expression. Figure 5 depicts the correlation of *ARID1A* allelic imbalance or the number of *ARID1A* loss-of-function mutations with ARID1A protein expression. All samples that harbored *ARID1A* allelic imbalance showed a loss of ARID1A protein expression. In addition, 14 of 15 samples that harbored multiple loss-of-function mutations showed a loss of ARID1A protein expression.

ARID1A protein expression was observed in 9 of 56 clear cell carcinoma samples (16.1%) with *ARID1A* loss-of-function mutations. Because we could not use serial sections for both immunohistochemical analysis in this study and target gene sequencing in the previous study, we validated the *ARID1A* mutation status of FFPE tissue samples in nine clear cell carcinoma samples with *ARID1A* truncating mutations. We macrodissected cancer cells, extracted DNA, and performed Sanger sequencing for *ARID1A* (Table 3). Although PCR was not successful in one sample due to poor DNA quality, we validated that seven FFPE samples harbored *ARID1A* loss-of-function mutations. In only one sample, the targeted *ARID1A* mutation was not detected by Sanger sequencing, probably because the mutation was in a subclonal state (MAF = 0.27).

**Table 3:** Validation of the *ARID1A* mutation status using FFPE samples from clear cell carcinomas with *ARID1A* loss-of-function mutations.

Patient	*ARID1A* mutation	MAF	ARID1A protein expression	Validation
OCCC_8	p.Y2148X	0.45	Positive	p.Y2148X
OCCC_23	p.Q393X	0.27	Positive	Normal
OCCC_50	p.R1461X	0.55	Positive	PCR failure
OCCC_61	p.Y395X	0.4	Positive	p.Y395X
OCCC_67	p.R1772X	0.31	Positive	p.R1772X
OCCC_74	p.Y1431X	0.46	Positive	p.Y1431X
OCCC_76	p.Q1454X/p.F1823fs	0.22/0.23	Positive	p.Q1454X/p.F1823fs
OCCC_77	p.Q393X	0.24	Positive	p.Q393X
OCCC_98	p.R727fs	0.31	Positive	p.R727fs

To confirm concordance of the ARID1A staining level between frozen sections and FFPE samples, we prepared frozen sections and FFPE samples from the same patient. ARID1A immunohistochemical staining of frozen sections was similar to that of FFPE samples in two clear cell carcinomas and ovarian endometriosis case (Supplementary Fig. 2).

Finally, we compared the clinicopathological features of ovarian clear cell carcinoma with *ARID1A* loss-of-function mutations to those of ovarian clear cell carcinoma without *ARID1A* mutations (Supplementary Table 3). Although the optimal rate of primary debulking surgery was marginally lower in the *ARID1A* loss-of-function mutation group than in the *ARID1A* wild-type group (*P* = 0.052), no significant differences in any clinicopathological characteristics, including prognosis, were observed (Supplementary Fig. 3).

## Discussion

Strong evidence for an association between ovarian endometriosis and ovarian clear cell and endometrioid carcinomas has been established in many studies^23–26^. In particular, there is epidemiological evidence that a personal history of endometriosis increases the risk of clear cell and endometrioid carcinomas^27–29^. Pathological studies have also demonstrated that atypical endometriosis merging between endometriosis and carcinoma exists in ovarian clear cell and endometrioid carcinoma cases^30,31^. In addition, there is accumulating molecular evidence linking endometriosis with clear cell carcinoma^7,14,26^. Wiegand et al. demonstrated that the loss of ARID1A caused by *ARID1A* loss-of-function mutations is observed in clear cell carcinoma and contiguous atypical endometriosis but not in distant endometriosis^7^. It is well known that *ARID1A* mutations are frequently detected in ovarian clear cell and endometrioid carcinomas but not in high-grade serous ovarian carcinomas^7,12,28,32^. These results suggest that *ARID1A* loss-of-function mutations are a driver event in endometriosis-associated ovarian cancer^6,7^. On the other hand, our recent studies clarified that cancer-associated genes such as *ARID1A*, *PIK3CA* and *KRAS* are frequently mutated not only in ovarian clear cell carcinoma but also in ovarian endometriosis^12,14^. Specifically, *ARID1A* loss-of-function mutations were detected in 7 of 54 ovarian endometriosis patients in our previous study^14^. Additionally, ovarian endometriosis samples harboring a single *ARID1A* loss-of-function mutation had mutations in oncogenes such as *PIK3CA* and *KRAS* and maintained benign conditions pathologically (Table 1)^14^. On the other hand, mutations in tumor suppressor genes, such as *PTEN*, *ATM*, and *TP53*, were not detected in these ovarian endometriosis samples. The significance of *ARID1A* mutations in the malignant transformation of ovarian endometriosis remains unclear.

Several studies have focused on ARID1A protein expression in endometriosis or ovarian cancer. Immunohistochemical analyses of ARID1A in ovarian cancer demonstrated that 0–40% of endometriosis lesions adjacent to ovarian cancer showed a loss of ARID1A protein expression, whereas all distant endometriosis lesions in ovarian cancer expressed ARID1A^7,17,19,21,33^. Similarly, several studies showed that ARID1A was expressed in almost all benign endometriosis lesions if ARID1A protein expression in stromal cells was correctly assessed as an internal positive control^17–22^. Although immunohistochemical staining and assessment protocols were not unified between studies, there was an obvious difference in ARID1A protein expression between benign endometriosis and endometriosis-associated ovarian cancer. These findings suggest that the loss of ARID1A protein expression might be an early driver event in the malignant transformation of ovarian endometriosis. However, the mutation status of *ARID1A* in endometriotic epithelial cells was not examined in these studies. Furthermore, the mechanism by which ARID1A protein expression is lost has not been sufficiently discussed. Wiegand et al. showed that 25% of clear cell carcinomas with loss-of-function mutations in one *ARID1A* allele retained ARID1A protein expression^7^. They also found that both mutant and wild-type alleles of *ARID1A* were expressed by using RNA sequencing data derived from nine clear cell carcinomas with *ARID1A* loss-of-function mutations. Based on these results, Wiegand et al. concluded that *ARID1A* could function as a haploinsufficient tumor suppressor. On the other hand, our study demonstrated that ARID1A protein expression was retained in 16% of clear cell carcinomas harboring *ARID1A* loss-of-function mutations (Fig. 4A). Moreover, ARID1A protein expression was retained in all benign endometriosis samples with *ARID1A* loss-of-function mutations. These findings are inconsistent with the concept of haploinsufficiency proposed by Wiegand et al.^7^. Our study also demonstrated that 14 of 15 samples that harbored multiple loss-of-function mutations showed a loss of ARID1A protein expression. In particular, all clear cell carcinoma samples harboring both *ARID1A* loss-of-function mutations and *ARID1A* allelic imbalance showed a loss of ARID1A protein expression. These findings suggest that the “two-hit” hypothesis can explain the cause of ARID1A loss in cancer cells^34–36^. In Knudson’s two-hit hypothesis^34^, germline mutation in tumor suppressor gene lead to a hereditary susceptibility to cancer and the inactivation of both alleles of tumor suppressor genes is essential to cause a phenotypic chance, leading to carcinogenesis. In other words, the “two-hit” hypothesis can explain why ARID1A protein expression was retained in all benign endometriosis samples with *ARID1A* loss-of-function mutations and a portion of clear cell carcinoma with *ARID1A* loss-of-function mutations. Taken together, these results suggest that the two-hit would be necessary for benign endometriosis with *ARID1A* heterozygous mutation to transform into malignant tumor.

Consistent with Wiegand et al.^7^, we also observed a portion of clear cell carcinomas without *ARID1A* mutations showed a loss of ARID1A protein expression, suggesting that epigenetic silencing, posttranscriptional and posttranslational regulation as well as genomic alterations might be important for the loss of ARID1A protein expression in clear cell carcinoma.

In this study, the sample size of ovarian endometriosis patients with *ARID1A* mutations was limited. It is necessary to assess ARID1A protein expression in ovarian endometriosis samples with *ARID1A* mutations in independent data sets. Although we used serial sections to assess *ARID1A* mutations and ARID1A protein expression in endometriosis, we could not extract DNA, RNA and protein from the same tissue simultaneously. There may be room for improvement not only in the number of samples but also in the extraction of DNA/RNA/protein for further study.

In conclusion, we clarified that ARID1A protein expression was retained in ovarian endometriosis samples harboring *ARID1A* loss-of-function mutations. The mechanism of ARID1A loss, which occurs specifically in endometriosis-associated ovarian cancer but not in ovarian endometriosis, is an important key for elucidating the pathogenesis of the malignant transformation of ovarian endometriosis.

## Material and methods

### Tissue samples

This study was performed in conformity with the Declaration of Helsinki and approved by the institutional ethics review boards of Niigata University, Niigata Chuo General Hospital, and the National Institute of Genetics. All patients provided written informed consent for the collection of samples and subsequent analyses.

We recruited 54 ovarian endometriosis patients for which whole-exome sequencing or target gene sequencing was conducted in our previous study^14^. We defined frameshift indels mutations, and nonsense mutations as *ARID1A* loss-of-function mutations. Then, we collected 16 frozen tissue section samples from seven patients with *ARID1A* mutations and 69 formalin-fixed paraffin-embedded (FFPE) samples from 47 patients without *ARID1A* mutations (Fig. 1). Frozen tissue samples were obtained from the same tissue blocks used for sequencing in our previous study^14^. Of these samples, one frozen tissue sample and six FFPE tissue samples were excluded from this study because there were no endometriotic epithelial cells in either the frozen tissue or FFPE sample.

We also enrolled 99 patients with ovarian clear cell carcinoma in this study to compare the association between ARID1A protein expression and the *ARID1A* mutation status with that in ovarian endometriosis. These clear cell carcinoma samples were already sequenced in our previous study^12^. Sixty-nine of 99 (69.7%) ovarian clear cell carcinoma samples harbored *ARID1A* mutations (Supplementary Table 2). Additionally, we prepared FFPE tissue sections from 99 clear cell carcinoma cases for immunohistochemical analysis (Fig. 3). We also prepared frozen tissue sections from two clear cell carcinomas to assess the concordance of ARID1A immunoreactivity between frozen tissue and FFPE samples in the same patient.

Hematoxylin and eosin-stained sections of all tissues used in this study were histologically reviewed by an experienced gynecologic pathologist (T.M.). All frozen tissue samples were cut from surgical specimens, embedded in Tissue-Tek O.C.T. compound (Sakura Finetek, Torrance, CA, USA) in a Tissue-Tek Cryomold (Sakura Finetek) and quickly frozen in liquid nitrogen as described in our previous study^14^.

### Immunohistochemical staining for ARID1A protein expression

Immunohistochemical analysis of ARID1A protein expression was performed for frozen tissue section and FFPE tissue section samples. A polyclonal rabbit anti-ARID1A antibody (HPA005456, Sigma-Aldrich, St. Louis, MO, USA) was used for immunostaining as a primary antibody. Frozen tissue sections (6 µm) and FFPE tissue sections (5 µm) were cut with a cryostat and a microtome, respectively. FFPE tissue sections were stained as previously described^37,38^. Briefly, after deparaffinization, antigen retrieval was carried out with Target Retrieval Solution (10 mM citrate buffer, pH 6.0; Dako, Tokyo, Japan) in a microwave for 20 min at 98 °C. Subsequently, the sections were incubated with the primary antibody (1:500 dilution) overnight and biotinylated secondary antibodies (Vector Laboratories, Burlingame, CA, USA) for 1 h, followed by incubation with ABC reagent (Dako) and 3,3′-diaminobenzidine (Sigma-Aldrich) for 3 min. Slides were counterstained with hematoxylin.

We fixed frozen tissue sections with 4% paraformaldehyde at 4 °C for 20 min followed by methanol at − 20 °C for 10 min. The immunohistochemical staining protocol after fixation was the same as the protocol for FFPE tissue sections.

We assessed normal nonepithelial cells, including endothelial cells, fibroblasts, and lymphocytes, as positive internal controls. The immunostaining was decided as positive if epithelial cells showed definite nuclear staining by two investigators (Y.N. and R.T.) The distribution of the percentage of positive cells showed bimodality as a previous study^39^. We evaluated samples with more than 80% positive cells as ARID1A positive and samples with under 20% positive cells as ARID1A loss (Supplementary Fig. 1). Samples in which normal cells in the stroma had no immunoreactivity were defined as having low antigenicity or low quality and excluded from the subsequent analysis.

### Validation of mutations by Sanger sequencing

To validate the mutation status of ovarian clear cell carcinoma FFPE samples, we prepared FFPE serial section following the one used for immunohistochemistry assay to perform Sanger sequencing per FFPE sample. We isolated tumor cells by needle macrodissection and extracted DNA using a QIAamp DNA FFPE Tissue Kit (QIAGEN Ltd., Manchester, UK) according to the manufacturer’s instructions.

We performed polymerase chain reaction (PCR) using a KAPA Taq EXtra HotStart ReadyMix PCR Kit, and the primers used are listed in Supplementary Table 4. We designed PCR primers using Primer3 software (https://bioinfo.ut.ee/primer3-0.4.0/). PCR products were purified and sequenced by GENEWIZ (Saitama, Japan).

### Statistical analysis

We conducted all standard statistical tests with the R program (https://www.r-project.org). We compared categorical variables between two groups by Fisher’s exact test and continuous variables between two groups by the Wilcoxon rank-sum test. Progression-free survival (PFS) and overall survival (OS) were estimated using the Kaplan–Meier method. Deviation in the mutant allele frequency (MAF) of the somatic mutation from 0.5 was assessed by a one-sided binomial test. A *P* value < 0.05 was considered allelic imbalance^40^.

## Supplementary information

Supplementary Information.
