# Supplementary material for: ARID1A protein expression is retained in ovarian endometriosis with ARID1A loss-of-function mutations: implication for the two-hit hypothesis
Source: Sci Rep. 2020 Aug 31;10:14260. doi: 10.1038/s41598-020-71273-7 (PMC7459315; doi:10.1038/s41598-020-71273-7)

**ARID1A protein expression is retained in ovarian endometriosis with *ARID1A* loss-of-function mutations: Implication for the two-hit hypothesis**

Nozomi Yachida^1^, Kosuke Yoshihara^1^, Kazuaki Suda^1^, Hirofumi Nakaoka^2,3^, Haruka Ueda^1^, Kentaro Sugino^1^, Manako Yamaguchi^1^, Yutaro Mori^1^, Kaoru Yamawaki^1^, Ryo Tamura^1^, Tatsuya Ishiguro^1^, Masanori Isobe^1^, Teiichi Motoyama^4^, Ituro Inoue^2^, Takayuki Enomoto^1^

1. Department of Obstetrics and Gynecology, Niigata University Graduate School of Medical and Dental Sciences, Niigata 951-8510, Japan

2. Human Genetics Laboratory, National Institute of Genetics, Mishima 411-8540, Japan

3. Department of Cancer Genome Research, Sasaki Institute, Sasaki Foundation, Chiyoda-ku 101-0062, Japan

4. Department of Molecular and Diagnostic Pathology, Niigata University Graduate School of Medical and Dental Sciences, Niigata 951-8510, Japan

**Supplementary Table 1. Comparison of clinicopathological features between ovarian endometriosis with *ARID1A* loss-of-function mutations and *ARID1A* wild-type.**

| Characteristics | *ARID1A* loss-of-function mutation (7) | *ARID1A* wild-type (47) | P-value |
| --- | --- | --- | --- |
| Age at operation (SD)* | 38.9 (6.8) | 39.7 (9.3) | P=0.835 |
|  |  |  |  |
|  |  |  |  |
| rASRM* |  |  |  |
| I | 0 | 0 | P=0.226 |
| II | 0 | 0 |  |
| III | 3 (43%) | 32 (86%) |  |
| IV | 4 (57%) | 15 (14%) |  |
|  |  |  |  |
| Pre-operation CA125 (U/ml)(SD)* | 599.0 (25) | 267.0 (31.6) | P=0.927 |
|  |  |  |  |
| Tumor size (cm)(SD)* | 5.9 (2.1) | 7.5 (3.5) | P=0.236 |
|  |  |  |  |
| Laterality** |  |  |  |
| Unilateral | 1 (14.3%) | 34 (72.3%) | P=0.006 |
| Bilateral | 6 (85.7%) | 13 (27.7%) |  |
| * Wilcoxon exact rank test |  |  |  |
| ** Fisher exact test |  |  |  |

**Supplementary Table 2. The association between *ARID1A* mutation status and ARID1A protein expression in 99 ovarian clear cell carcinomas.**

| Samples | *ARID1A* mutation | Mutant allele frequency | Allelic imbalance | ARID1A protein expression |
| --- | --- | --- | --- | --- |
| OCCC_1 | splicing variant | 0.49 | - | NA |
| OCCC_2 | p.R1276X / p.L1866fs / p.P1867fs / p.S1868fs | 0.33 / 0.33 /0.30 / 0.30 | - | loss |
| OCCC_3 | p.S584fs | 0.94 | + | loss |
| OCCC_4 | p.G272fs | 0.72 | + | loss |
| OCCC_5 | none | - | - | NA |
| OCCC_6 | none | - | - | positive |
| OCCC_7 | p.Y422_G423delinsX | 0.38 | - | loss |
| OCCC_8 | p.Y2148X | 0.45 | - | positive |
| OCCC_9 | p.S1985P | 0.54 | - | positive |
| OCCC_10 | p.T979fs / p.F1118fs | 0.45 / 0.41 | - | loss |
| OCCC_11 | p.R2158X | 0.89 | + | loss |
| OCCC_12 | none | - | - | positive |
| OCCC_13 | p.W1670X | 0.72 | + | loss |
| OCCC_14 | none | - | - | loss |
| OCCC_15 | p.I2185fs | 0.45 | - | NA |
| OCCC_16 | p.R1276X / p.G1630fs | 0.43 / 0.34 | - | NA |
| OCCC_17 | p.Y1285fs | 0.52 | - | loss |

**Supplementary Table 2. Continued**

| Samples | *ARID1A* mutation | Mutant allele frequency | Allelic imbalance | ARID1A protein expression |
| --- | --- | --- | --- | --- |
| OCCC_18 | p.E1776X | 0.47 | - | NA |
| OCCC_19 | none | - | - | positive |
| OCCC_20 | none | - | - | NA |
| OCCC_21 | p.Q581X | 0.90 | + | loss |
| OCCC_22 | p.T2138fs | 0.44 | - | NA |
| OCCC_23 | p.Q393X | 0.27 | - | positive |
| OCCC_24 | p.Q611X / p.P726fs | 0.38 / 0.42 | - | loss |
| OCCC_25 | none | - | - | positive |
| OCCC_26 | none | - | - | loss |
| OCCC_27 | p.N917fs | 0.57 | - | loss |
| OCCC_28 | none | - | - | positive |
| OCCC_29 | p.Q585X | 0.66 | + | NA |
| OCCC_30 | none | - | - | positive |
| OCCC_31 | none | - | - | NA |
| OCCC_32 | p.E2058X | 0.41 | - | loss |
| OCCC_33 | none | - | - | loss |
| OCCC_34 | none | - | - | loss |

**Supplementary Table 2. Continued**

| Samples | *ARID1A* mutation | Mutant allele frequency | Allelic imbalance | ARID1A protein expression |
| --- | --- | --- | --- | --- |
| OCCC_35 | none | - | - | positive |
| OCCC_36 | splicing variant | 0.39 | - | positive |
| OCCC_37 | p.S617X | 0.27 | - | NA |
| OCCC_38 | p.E1006X / p.N1192fs | 0.32 / 0.59 | + | loss |
| OCCC_39 | none | - | - | positive |
| OCCC_40 | p.M1215fs | 0.7 | + | loss |
| OCCC_41 | none | - | - | positive |
| OCCC_42 | p.Q2039fs | 0.38 | - | loss |
| OCCC_43 | none | - | - | positive |
| OCCC_44 | none | - | - | NA |
| OCCC_45 | p.I1954fs / p.Q2176fs | 0.50 / 0.54 | - | loss |
| OCCC_46 | p.A2175fs | 0.2 | - | loss |
| OCCC_47 | p.E896X / p.E1799fs | 0.41 / 0.26 | - | loss |
| OCCC_48 | p.Y2031X | 0.99 | + | loss |
| OCCC_49 | none | - | - | positive |
| OCCC_50 | p.R1461X | 0.55 | - | positive |
| OCCC_51 | none | - | - | positive |

**Supplementary Table 2. Continued**

| Samples | *ARID1A* mutation | Mutant allele frequency | Allelic imbalance | ARID1A protein expression |
| --- | --- | --- | --- | --- |
| OCCC_52 | none | - | - | positive |
| OCCC_53 | p.G108fs | 0.52 | - | loss |
| OCCC_54 | none | - | - | loss |
| OCCC_55 | none | - | - | loss |
| OCCC_56 | none | - | - | positive |
| OCCC_57 | p.Q1990X / p.Q920fs | 0.44 / 0.37 | - | loss |
| OCCC_58 | p.Q393X / p.L1753fs | 0.36 / 0.38 | - | loss |
| OCCC_59 | splicing variant/p.A1682fs | 0.48 / 0.38 | - | loss |
| OCCC_60 | splicing variant/p.S138X | 0.64 / 0.17 | - | loss |
| OCCC_61 | p.Y395X | 0.4 | - | positive |
| OCCC_62 | p.Q1403X / p.G284fs | 0.28 / 0.38 | - | loss |
| OCCC_63 | p.M1224fs | 0.48 | - | loss |
| OCCC_64 | p.Q507fs | 0.67 | + | NA |
| OCCC_65 | p.G1847fs | 0.49 | - | loss |
| OCCC_66 | p.Q405X/p.R1989X | 0.34/0.60 | + | loss |
| OCCC_67 | p.R1989X | 0.31 | - | positive |
| OCCC_68 | p.S1184fs /p.V1982fs | 0.26 / 0.56 | - | loss |

**Supplementary Table 2. Continued**

| Samples | *ARID1A* mutation | Mutant allele frequency | Allelic imbalance | ARID1A protein expression |
| --- | --- | --- | --- | --- |
| OCCC_69 | p.E1687X / p.G1630fs | 0.43 / 0.32 | - | loss |
| OCCC_70 | none | - | - | positive |
| OCCC_71 | p.Q502X | 0.31 | - | loss |
| OCCC_72 | p.I908fs | 0.67 | + | loss |
| OCCC_73 | none | - | - | positive |
| OCCC_74 | p.Y1431X | 0.46 | - | positive |
| OCCC_75 | p.Y823fs | 0.18 | - | loss |
| OCCC_76 | p.Q1454X / p.F1823fs | 0.22 / 0.23 | - | positive |
| OCCC_77 | p.Q393X | 0.24 | - | positive |
| OCCC_78 | p.Q548fs | 0.8 | + | loss |
| OCCC_79 | p.E1643X | 0.66 | + | loss |
| OCCC_80 | p.E1818X | 0.76 | + | loss |
| OCCC_81 | p.Q199X / p.Y518X | 0.35 / 0.69 | + | loss |
| OCCC_82 | none | - | - | positive |
| OCCC_83 | p.F1859fs | 0.42 | - | loss |
| OCCC_84 | p.L714fs | 0.86 | + | loss |
| OCCC_85 | p.I1954fs | 0.46 | - | NA |

**Supplementary Table 2. Continued**

| Samples | *ARID1A* mutation | Mutant allele frequency | Allelic imbalance | ARID1A protein expression |
| --- | --- | --- | --- | --- |
| OCCC_86 | none | - | - | positive |
| OCCC_87 | p.T1003fs | 0.33 | - | loss |
| OCCC_88 | none | - | - | positive |
| OCCC_89 | p.S1153fs | 0.91 | + | loss |
| OCCC_90 | none | - | - | positive |
| OCCC_91 | p.Y1324fs | 0.26 | - | loss |
| OCCC_92 | p.Q372X / p.R750X | 0.52 / 0.30 | - | loss |
| OCCC_93 | splicing variant | 0.76 | + | loss |
| OCCC_94 | p.L2279fs | 0.84 | + | loss |
| OCCC_95 | p.W1073X | 0.59 | - | loss |
| OCCC_96 | p.Q566X | 0.61 | + | loss |
| OCCC_97 | splicing variant | 0.07 | - | loss |
| OCCC_98 | p.R727fs | 0.31 | - | positive |
| OCCC_99 | p.T292fs | 0.53 | - | loss |

**Supplementary Table 3. Comparison of clinicopathological features between ovarian clear cell carcinoma with *ARID1A* loss-of-function mutations and *ARID1A* wild-type.**

| Characteristics | *ARID1A* loss-of-function mutation (64) | *ARID1A* wild-type (30) | P-value |
| --- | --- | --- | --- |
| Age at diagnosis (SD)* | 55.6 (10.0) | 53.8 (11.7) | 0.365 |
|  |  |  |  |
| FIGO_stage* |  |  |  |
| I | 39 (61%) | 19 (63%) | 0.624 |
| II | 7 (11%) | 5 (17%) |  |
| III | 13 (20%) | 5 (17%) |  |
| IV | 5 (8%) | 1 (3%) |  |
|  |  |  |  |
| Pre-operation CA125 (U/ml)(SD)* | 665.9 (1742.8) | 812.7 (1723.7) | 0.935 |
|  |  |  |  |
| Tumor size (cm)(SD)* | 12.9 (4.2) | 12.4 (4.1) | 0.864 |
|  |  |  |  |
| Residual disease |  |  |  |
| Optimal surgery (≦1cm)** | 56 (88%) | 30 (100%) | 0.052 |
| Suboptimal surgery (>1cm) | 8 (12%) | 0 |  |
| * Wilcoxon exact rank test |  |  |  |
| ** Fisher exact test |  |  |  |

**Supplementary Table 4. The list of PCR primers by which we performed validation of mutation status of 9 ovarian clear cell carcinomas with *ARID1A* loss of function mutations.**

|  | **Forward** | **Reverse** |
| --- | --- | --- |
| OCCC_8 | 5'-ATACCCCGAGAGCATTTGCC-3' | 5'-CAGTACCACAGCCATCTCCC-3' |
| OCCC_23 | 5'-GAGGCGGGTCAGTTGACTTA-3' | 5'-GTATGGCTGCCCTGGGTAC-3' |
| OCCC_50 | 5'-GGCACGAAGGGGAGATGTAC-3' | 5'-CAACCTCAGCTGATGCCTGT-3' |
| OCCC_61 | 5'-GAGGCGGGTCAGTTGACTTA-3' | 5'-GTATGGCTGCCCTGGGTAC-3' |
| OCCC_67 | 5'-AGAGCCACCGGAACATCAAG-3' | 5'-CCACTTTGTTGCAGCTCACC-3' |
| OCCC_74 | 5'-GGCACGAAGGGGAGATGTAC-3' | 5'-CAACCTCAGCTGATGCCTGT-3' |
| OCCC_76 | 5'-GGCACGAAGGGGAGATGTAC-3' | 5'-CAACCTCAGCTGATGCCTGT-3' |
| OCCC_76 | 5'-GGACAAGCCAGCTTCAGAGA-3' | 5'-CTGTTGTCCCTGGTGTACCC-3' |
| OCCC_77 | 5'-GAGGCGGGTCAGTTGACTTA-3' | 5'-GTATGGCTGCCCTGGGTAC-3' |
| OCCC_98 | 5'-TGGAACCTGTTGGCTGGATC-3' | 5'-ATGTCTGCCCTAGCTCCCTG-3' |

**Supplementary Figure 1. Distribution of the percentage of ARID1A positive cells in ovarian clear cell carcinoma.**

**The distribution of the percentage of positive cells showed bimodality.**


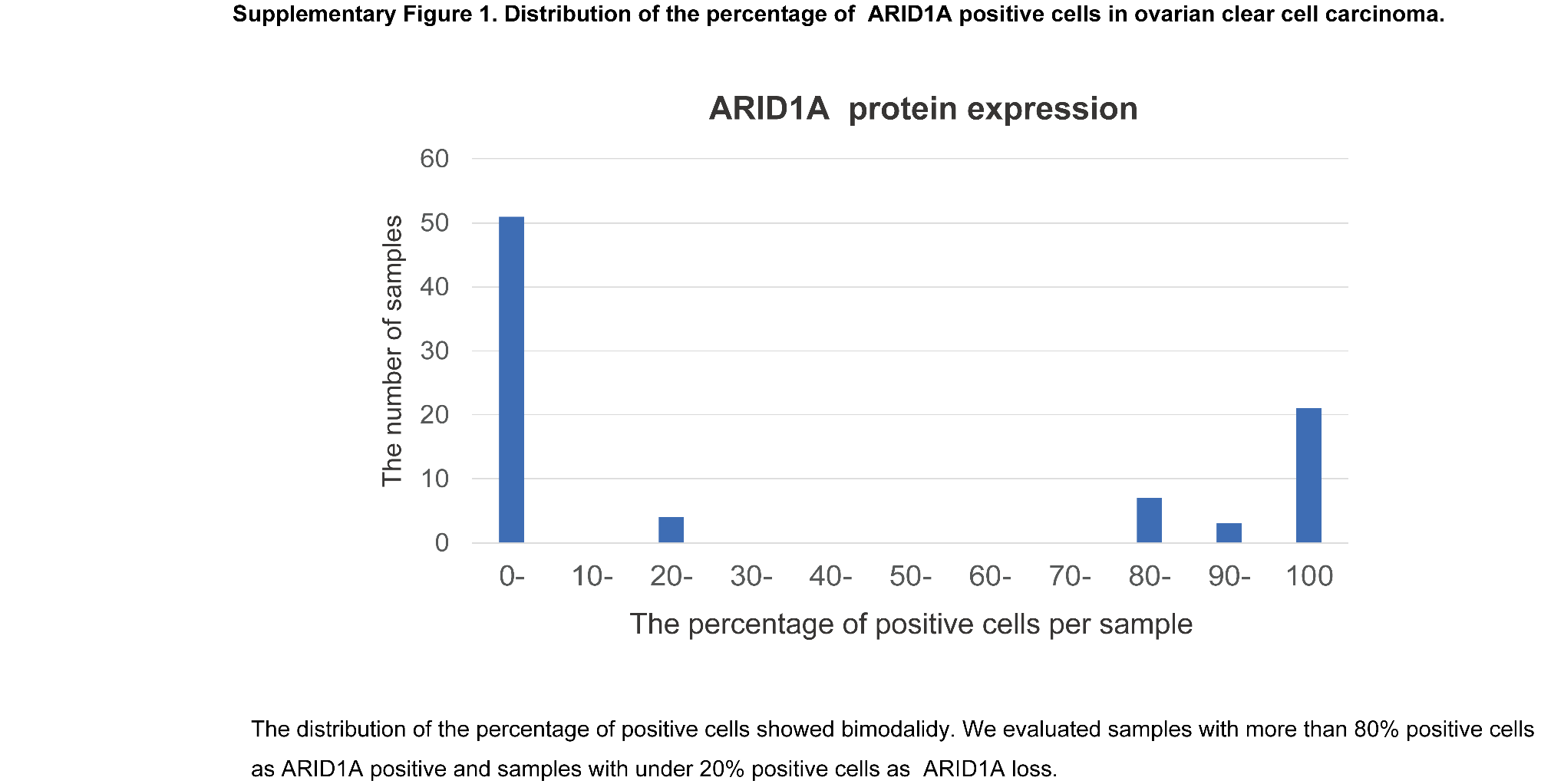


We evaluated samples with more than 80% positive cells as ARID1A positive and samples with under 20% positive cells as ARID1A loss.

**Supplementary Figure 2. Concordance of the staining level between FFPE and frozen section (FS) in the same case.**


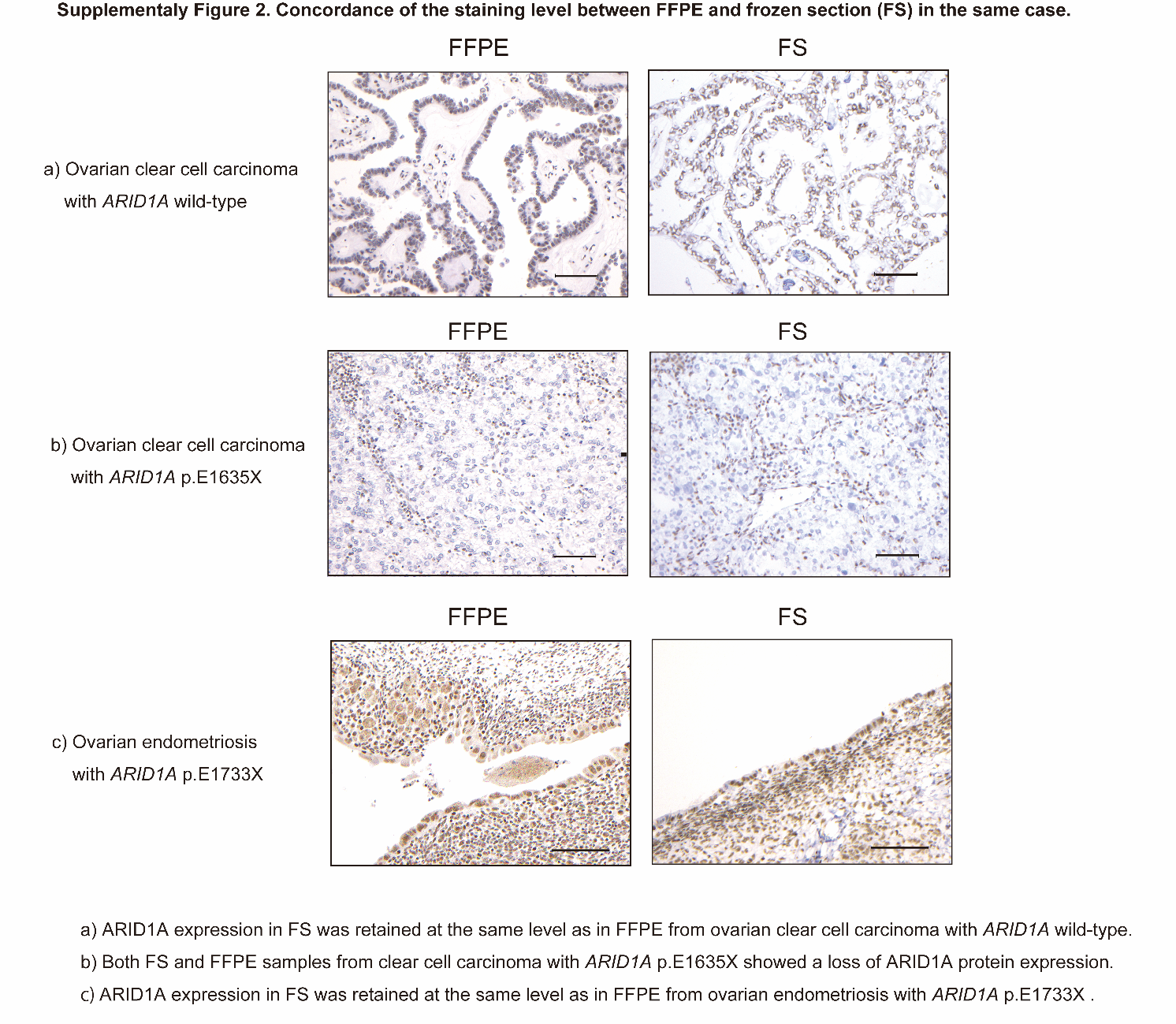


a) ARID1A expression in FS was retained at the same level as in FFPE from ovarian clear cell carcinoma with *ARID1A* wild-type.

b) Both FS and FFPE samples from clear cell carcinoma with *ARID1A* p.E1635X showed a loss of ARID1A protein expression.

c) ARID1A expression in FS was retained at the same level as in FFPE from ovarian endometriosis with *ARID1A* p.E1733X .

**Supplementary Figure 3. Kaplan-Meier survival curves of ovarian clear cell carcinoma patients with *ARID1A* loss-of-function mutations and *ARID1A* wild-type.**


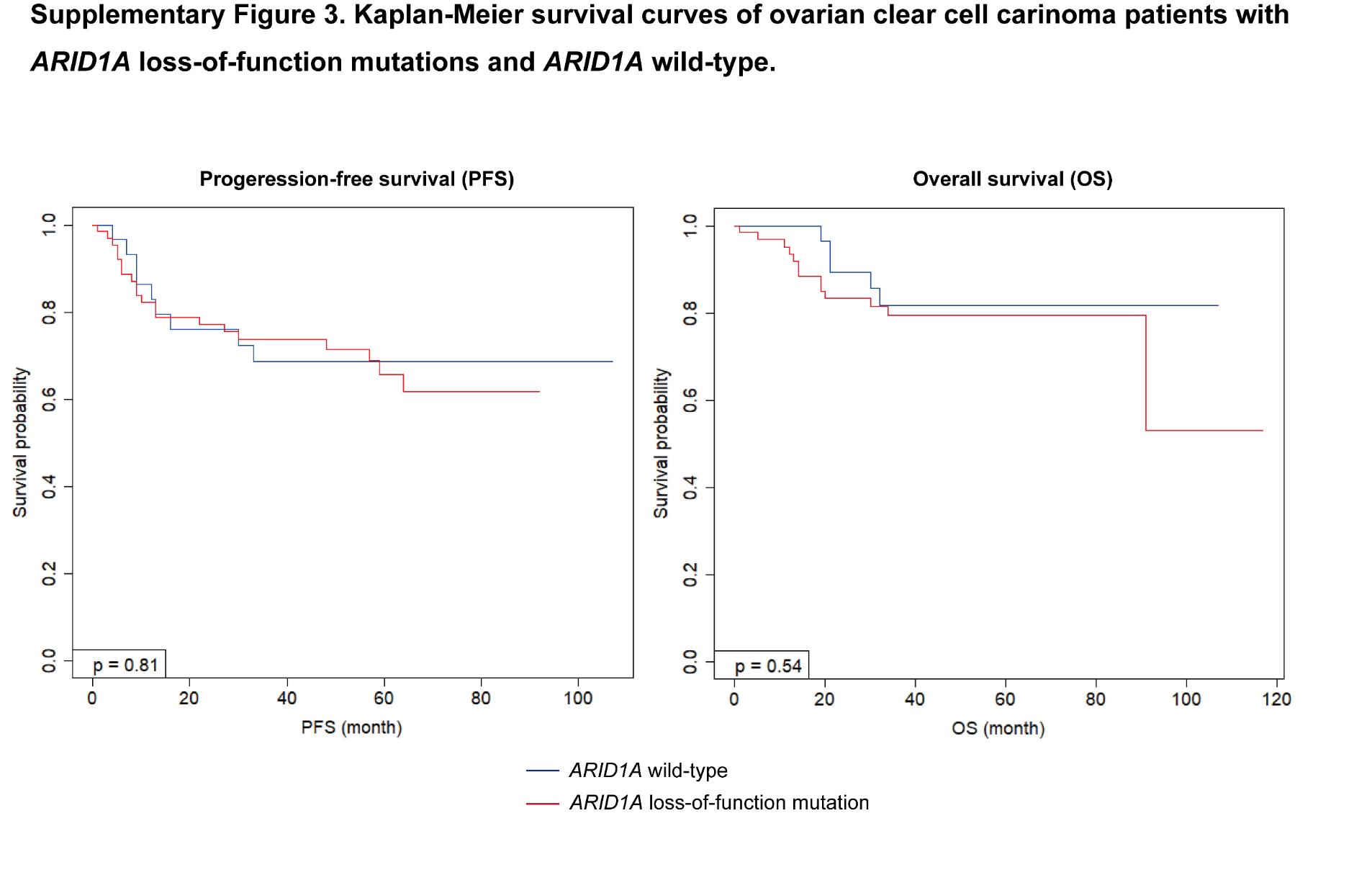

Supplement: Supplementary file 1 — Supplementary Information. [file 41598_2020_71273_MOESM1_ESM.docx]
